# Factors That Can Undermine the Psychological Benefits of Coastal Environments: Exploring the Effect of Tidal State, Presence, and Type of Litter

**DOI:** 10.1177/0013916515592177

**Published:** 2015-07-03

**Authors:** Kayleigh J. Wyles, Sabine Pahl, Katrina Thomas, Richard C. Thompson

**Affiliations:** 1 School of Psychology, Plymouth University, Drake Circus, Plymouth, UK; 2 Plymouth Marine Laboratory, Prospect Place, Plymouth, Devon, UK; 3 School of Marine Science and Engineering, Plymouth University, Drake Circus, Plymouth, UK

**Keywords:** marine debris, attention restoration theory, affect, connectedness to nature, disrespect for nature, restoration likelihood

## Abstract

The beneficial effects of blue environments have been well documented; however, we do not know how marine litter might modify these effects. Three studies adopted a picture-rating task to examine the influence of litter on preference, perceived restorative quality, and psychological impacts. Photographs varied the presence of marine litter (Study 1) and the type of litter (Studies 2 and 3). The influence of tide and the role of connectedness were also explored. Using both quantitative and qualitative methods, it was shown that litter can undermine the psychological benefits that the coast ordinarily provides, thus demonstrating that, in addition to environmental costs of marine litter, there are also costs to people. Litter stemming from the public had the most negative impact. This research extends our understanding of the psychological benefits from natural coastal environments and the threats to these benefits from abundant and increasing marine litter.

## Introduction

Natural environments provide a range of psychological benefits to their visitors, especially blue environments, such as coastlines (Hipp & Ogunseitan, 2011; Laumann, Gärling, & Stormark, 2001; M. P. White, Pahl, Ashbullby, Herbert, & Depledge, 2013; M. P. White et al., 2010). Unfortunately, human activities can harm these environments, which may in turn have detrimental impacts on our experiences. Marine litter, manufactured solid waste material that enters the marine environment, is a worldwide problem that dramatically transforms the environment (Galgani et al., 2010). Such litter contaminates habitats from the poles to the equator and from the shoreline to the deep sea and is commonly found on the coast (Obbard et al., 2014; Thompson, Moore, vom Saal, & Swan, 2009; Woodall et al., 2014). Many of the materials are extremely slow to degrade and thus are likely to remain in the ocean for hundreds of years (Kershaw, Katsuhiko, Lee, Leemseth, & Woodring, 2011). Consequently, this human-made waste continues to have a prolonged negative impact on the environment and its inhabitants through processes such as ingestion, entanglement, and chemical contamination from eating those materials (Gall & Thompson, 2015; Kershaw et al., 2011). We have considerable knowledge about the benefits of *clean* natural environments, and recent literature has emphasized that blue (aquatic) and green (terrestrial) environments may differ in important aspects (M. P. White et al., 2010). However, we know less about how the presence of litter may interfere with these benefits, especially marine litter in coastal environments. This article consequently investigates the psychological impact of marine litter. First, the literature regarding the psychological impacts of clean and littered environments is summarized. Three studies are then reported: Study 1 explores whether the presence of rubbish and tidal state influences the perceived restorative quality of coastal environments; Studies 2 and 3 systematically investigate the influence of different types of litter on preference, affect, and restoration likelihood.

### Literature Review

Clean natural environments, especially those along the coast, have been found to be preferred environments, and a number of positive impacts on visitors have been demonstrated on affect and perceived restorative quality. Coastal environments are typically noted as being aesthetically pleasing, with people willing to spend considerably more money and time in these environments than for other alternatives (Herzog, 1985; Luttik, 2000; Nanda, Eisen, & Baladandayuthapani, 2008; Wynveen, Kyle, & Sutton, 2010). Within the psychological literature, it is proposed that natural environments are beneficial because they accommodate four restorative properties. The attention restoration theory (ART; Kaplan & Kaplan, 1989) states that environments that can give a sense of *being away* (psychological distance from everyday stressors), *fascination* (the ability to capture involuntary attention), *extent* (the richness of the environment), and *compatibility* (the ability to fulfill a person’s intention) will facilitate positive experiences. Coastlines have been shown to have these perceived restorative qualities (Hipp & Ogunseitan, 2011; M. P. White et al., 2010), and in turn, have been found to yield benefits, such as changes in affect (e.g., feeling happier) and psychological restoration (feeling revitalized, calm, and refreshed; Ashbullby, Pahl, Webley, & White, 2013; M. P. White, Pahl, et al., 2013; M. P. White et al., 2010).

While coastlines have these perceived restorative qualities and can provide benefits to their visitors, the condition of the environment can vary dramatically, which can influence people’s experiences. For instance, weather, a dynamic feature for all environments, can have a strong influence (M. P. White, Cracknell, Corcoran, Jenkinson, & Depledge, 2013). Coastal environments are also transformed by the tide. In the United Kingdom, as the water retreats, substantial areas of the intertidal area become exposed. This exposure consequently changes the appearance of the shoreline, varying the level of intertidal area that is visible and potentially the restorative qualities of the environment. Yet, to the authors’ knowledge, no research has explicitly explored whether such exposure influences individuals’ experiences.

Anthropogenic impacts can also influence the condition of an environment. The recreational ecology and leisure literature have examined how recreational visits impact the environment (“recreational carrying capacity”) and its visitors (“social carrying capacity”; Anderson & Brown, 1984). Although many papers focus on crowding (e.g., Stankey & Manning, 1986), others have found that depreciative behavior (such as littering) can be especially negative. For instance, visitors disliked and were less accepting of litter than other recreational impacts (Anderson & Brown, 1984; Eder & Arnberger, 2012; Shafer & Hammitt, 1995). This effect was demonstrated by Budruk and Manning (2006) who carried out a study using photographs that systematically varied the extent of litter and graffiti present. A large sample of visitors rated scenes more acceptable if no litter/graffiti or only a small amount was present. As well as being disliked, litter has also been stated as a reason to not visit a particular site (Anderson & Brown, 1984; Ballance, Ryan, & Turpie, 2000; Tudor & Williams, 2006; World Health Organization [WHO], 2003). While the difference seems subtle, focusing respondents on the presence of litter is psychologically very different from asking them to rate how pleasant natural scenes would be to visit (without explicitly mentioning the potential presence of litter).

Even when not emphasizing litter as the focus of the study, its presence has been reflected in ratings. Wilson, Robertson, Daly, and Walton (1995) digitally manipulated waterscape pictures to either be clean or have visual cues of degradation (including not only litter items but also surface foam and algal bloom). They found that scenes indicating environmental degradation were less liked and were less likely to be picked for hypothetical future visits compared with the pristine alternatives. Unlike these previous studies that have tended to focus on acceptability and preferences, another study focused directly on the psychological and physiological effects of clean versus degraded green environments (Pretty, Peacock, Sellens, & Griffin, 2005). Scenes that included degraded features such as damaged trees, burnt-out cars, and litter were found to not be as effective in improving mood or reducing blood pressure as the clean alternative scenes. These findings are initial evidence that litter (along with other degraded features) can reduce the psychological benefits a pristine environment usually offers. However, the degradation features tested here were rather extreme, and the findings do not address blue environments.

The level to which an environment is beneficial and litter is harmful may be influenced by individuals’ emotional bonds with nature. For instance, Mayer, Frantz, Bruehlman-Senecal, and Dolliver (2009) found that individuals who were more connected to nature, that is, had a stronger emotional bond to the natural world, received greater benefits from nature visits than did those who were less connected. However, the influence of connectedness on a person’s experience of a littered environment has yet to be investigated. Reflecting on the place attachment literature that focuses on identity and dependency on specific sites (Kyle, Graefe, Manning, & Bacon, 2004), the conclusions about this relationship are mixed. Some studies have found that individuals with a greater place attachment to a particular site are more sensitive to litter than are those with a weaker bond to nature (Kyle et al., 2004), others suggest that those with a stronger bond have a greater adaptive capacity and thus are able to overlook this negative impact (Marshall, Tobin, Marshall, Gooch, & Hobday, 2013), whereas opposing work has found that litter is perceived negatively regardless of individuals’ initial bonds (Eder & Arnberger, 2012; D. D. White, Virden, & van Riper, 2008). These studies mainly focus on individuals’ perceptions of the condition of a (typically green) environment, thus it is still unknown how (or if) the psychological impact of experiencing a littered environment is influenced by an individual’s initial bond.

While Budruk and Manning (2006) systematically examined the impacts of differing quantities of litter in relation to carrying capacity and found that people dislike the smallest amount of litter, no research to date has looked at the impact of *type* of litter. The recreation literature appears to focus solely on visitor-caused litter because of its interest in managing natural parks. While visitors also contribute to coastal litter, there are other sources marine litter has been attributed to. Roughly 40% of marine litter found on the U.K. coast are thought to consist of items that are accidently or deliberately left on the beach or carried there by winds and rivers, including drink bottles, sweet and crisp wrappers, and barbeque remains (termed as “public-litter”; Marine Conservation Society [MCS], 2012). The second most common source is classed as “non-sourced,” as these items are unable to be identified as they are too small and/or damaged (MCS, 2012). The third most common category is “fishing-litter,” which typically includes pieces of fishing crates, fishing rope and lines, and industrial rubber gloves (MCS, 2012). To represent typical experiences with a littered environment (strengthening ecological validity), studies manipulating litter accordingly should represent these more commonly found categories. This approach differs from the earlier studies (Pretty et al., 2005; Wilson et al., 1995) that used highly salient but, in reality, less commonly observed items such as burnt-out cars or floating tires. As highlighted by Pretty and colleagues (2005), it is also important to consider natural debris that does not contribute to marine litter as an environmental issue but may still reduce the pristineness of a setting. For example, drift (dislodged) seaweed is naturally found on the shore but can be associated with negative impacts on individuals due to its unattractive smell and aesthetics (WHO, 2003). Thus, further research is needed to examine in greater detail the impacts “debris” (we use this term to encompass both marine litter and drift seaweed) has on individuals; specifically, whether there are differences in impact between different types of debris and why. This approach would help further emphasize the need to address marine litter, as well as to indicate whether specific sources of litter should be targeted.

### Present Studies

Whereas prior research has studied the negative impacts of litter on individuals, which may or may not be influenced by their emotional bonds to nature, no research to date has a) examined this within the marine context, b) systematically manipulated the presence and type of the rubbish, and c) focused solely on litter. Three studies were conducted to examine the impacts of varying states of the coastal environment on individuals (in terms of preference, affect, and restoration likelihood), as well as uniquely evaluating the environment according to ART’s restorative properties, with a particular focus on marine litter. Using a laboratory approach, we systematically manipulated photographical stimuli, which were rated by three different participant samples. Study 1 manipulated tidal state (high or low tide) and the presence of marine litter (clean or littered), which were then rated according to ART’s key properties (“perceived restorative quality”) and preference. Controlling for tidal state, Studies 2 and 3 then examined the effect of types of debris (including natural sea weed) by exposing people to four conditions: clean, seaweed, public-litter, and fishing-litter. Study 2 used a quantitative approach asking participants to rate images according to preference, affect, and restoration likelihood; Study 3 focused primarily on qualitative responses justifying the same ratings. The latter two studies also explored the role of connectedness to nature.

Overall, the aim of these three studies was to answer two primary research questions:

**Research Question 1:** Does the presence of marine litter undermine the benefits coastal environments are normally perceived to provide, and how does this relate to ART’s key properties?
**Research Question 2:** Do these psychological impacts differ according to type of debris (drift seaweed, public-litter, and fishing-litter), and if so, why?

In addition to these two key questions, this article also addresses three secondary questions: Does tide (a naturally varying state of the environment) influence the restorative potential of the coast; does initial connectedness to nature influence people’s experiences of these environments; and finally, to embed the coastal findings, how do these ratings compare with other (clean) environment types that have been used in prior research?

## Study 1: Do Litter and Tide Have an Impact?

### Method

#### Experimental stimuli

The experimental stimuli consisted of 12 individual photographs taken on British sandy beaches under dry weather conditions (picture quality was further controlled using computer software). A total of four within-subject conditions were created: clean high tide, clean low tide, littered high tide, and littered low tide. Litter items found on-site that are commonly found on the U.K. coastline were used (MCS, 2012) and later edited in (or out) of photographs using computer software, so that the background was identical to the respective clean tidal state. Litter stimuli consisted of 10 to 21 items that took up approximately 7% of the total image. Twelve additional photographic stimuli of coastlines were used as filler stimuli to reduce the salience of these manipulations but were not included in the analysis.

#### Participants and design

The sample consisted of 40 undergraduates from Plymouth University’s School of Psychology Participation Pool who were given course credit for their participation. The majority of the sample were women (90%) with an average age of 21 years (*SD* = 4.71).

A 2 (tide: high, low) × 2 (litter: clean, littered) within-subject design was applied, whereby each participant rated all 24 photographs. Participants received the same pre-set order, which was initially randomly generated, then adjusted to avoid similar conditions following one another.

#### Measures

Each image was rated according to perceived restorative quality based on ART (Kaplan & Kaplan, 1989). Items addressed being away (*that is a place which is away from everyday demands and where I would be able to relax and think about what interests me*), fascination (*that place is fascinating; it is large enough for me to discover and be curious about things*), extent (*that is a place which is very large, with no restrictions to movements; it is a world of its own*), and compatibility (*in that place it is very easy to orient and move around so that I can do what I like*; Berto, 2005). The item for coherence was omitted due to the debate on its appropriateness of use (M. P. White et al., 2010). A 10-point scale was used from *not at all* (1) to *very much* (10). When items were averaged and combined into a scale of perceived restorative quality, this showed good reliability in each within-subject condition (Cronbach’s αs > .96). Images were also rated according to preference, with participants rating *how likely would it be that you chose to spend time here* on the scale as previously indicated here.

#### Procedure and analysis

Once seated in front of a computer monitor, participants gave informed consent, provided demographic information, and continued on to the rating task. Images were displayed individually in the middle of the computer monitor, covering roughly 75% of the screen. Participants were required to rate each picture on the five rating scales, which appeared one by one underneath the images. Participants first completed a trial run with 3 images (not included in the analysis), then proceeded onto the 24 test images (12 experimental stimuli and 12 filler images). On completion of the study, participants were thanked for their participation and debriefed.

For this and the later studies, some data were non-normally distributed, thus both non-parametric and parametric tests were used for the following analysis, with the latter reported unless conclusions differed. For each of the four conditions, average responses were calculated for perceived restorative quality and preference. The main analysis consisted of 2 (tide: high, low) × 2 (litter: clean, littered) repeated ANOVAs. To further investigate any interactions, simple effects analyses used paired *t* tests while applying Bonferroni correction. The main analyses were not statistically related to gender (*p* = .30 for perceived restorative quality and *p* = .37 for preference) or age (*p* = .17 and *p* = .07, respectively), and thus these were not considered further.

### Results

For both perceived restorative quality and preference, the same pattern emerged (see Table 1). Beaches during low tide were perceived to have a higher restorative quality and were more highly preferred than were those during high tide. Moreover, littered beaches were seen to have lower restorative quality and were less preferred than were the clean alternatives (see Table 1 for the inferential statistics). Finally, the interaction between tidal state and presence of litter was statistically significant for perceived restorative quality. Even though ratings of restorative quality did decline during high tide compared with low tide, ratings were considerably worse when litter was present in either tidal state. Perceived restorative quality was much lower for environments with litter during both low tide, *t*(39) = 8.48, *p* < .001, *d* = 1.38 (large effect), and high tide, *t*(39) = 8.36, *p* < .001, *d* = 1.18 (large effect), compared with the clean alternatives.

**Table 1.** Participants’ Average Ratings (and Standard Deviations) of Perceived Restorative Quality, Preference, and Associated Inferential Statistics in Study 1 (*n* = 40).

		Tidal condition			
	Litter condition	Low tide	High tide	Inferential statistics	
Perceived restorative quality	Clean	7.91 (1.34)	7.13 (1.28)	Main effect of tide:	*F*(1, 39) = 37.63, *p* < .001, $\eta_{p}^{2}$ = .49 (large effect size)
	Littered	5.56 (1.71)	5.20 (1.63)	Main effect of litter:	*F*(1, 39) = 78.31, *p* < .001, $\eta_{p}^{2}$ = .67 (large effect size)
				Interaction (Litter × Tide):	*F*(1, 39) = 6.81, *p* = .01, $\eta_{p}^{2}$ = .15 (small effect size)
Preference	Clean	7.95 (1.59)	7.08 (1.48)	Main effect of tide:	*F*(1, 39) = 35.16, *p* < .001, $\eta_{p}^{2}$ = .47 (large effect size)
	Littered	4.97 (1.88)	4.51 (1.81)	Main effect of litter:	*F*(1, 39) = 97.81, *p* < .001, $\eta_{p}^{2}$ = .72 (large effect size)
				Interaction (Litter × Tide):	*ns*

*Note.* The scale for perceived restorative quality and overall preference ranged from 1 = *not at all* to 10 = *very much. ns* = non-significant.

## Study 2: Does Type of Litter Have an Impact?

Study 1 demonstrated that the restorative quality of an environment and preference ratings differed depending on tidal state and presence of litter. In particular, both were rated lower during high tide and when litter was present. Litter in particular considerably reduced the restorative properties the clean coast was perceived to provide. However, there were some methodological shortcomings, and the type of litter was unspecified. Consequently, Study 2 increased the sample size, distinguished between different types of litter and natural debris, and included other environment types. Keeping tidal state low throughout, Study 2 investigated whether public-litter, fishing-litter, and drift seaweed had similar impacts and compared these with other (clean) natural environments. Finally, Study 2 also sought to answer whether an individual’s initial connectedness to nature influences his or her experiences of these environments.

### Method

#### Experimental stimuli

**Figure 1.**
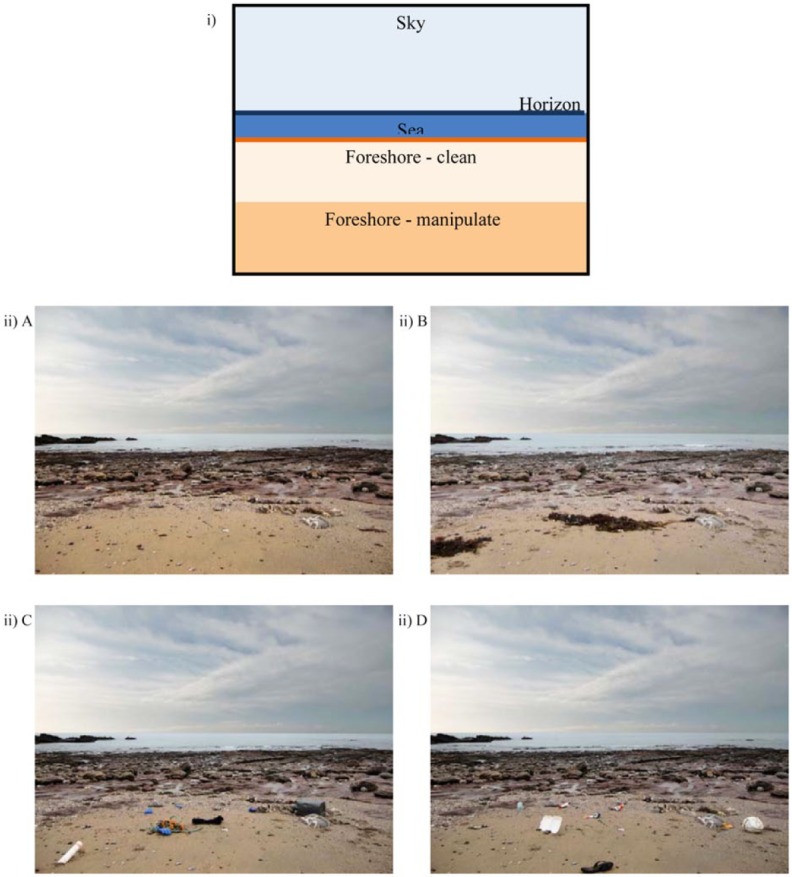
The format for (i) each individual image for the experimental conditions and (ii) an example of the four experimental conditions: (A) clean, (B) seaweed, (C) fishing-litter, and (D) public-litter used in Studies 2 and 3 (*n*s = 79 and 19, respectively).

All of the photographical stimuli consisted of the same format and backdrop (see Figure 1) and were taken on dry days under clement weather conditions. Photographs were taken during low tide (as rated as more restorative in Study 1) at numerous rocky shore sites. By positioning the camera accordingly, the images were taken from a perspective as though the viewer was sitting on the shore looking out to sea. Lighting, weather conditions, and overall picture quality were further matched using computer software.

The conditions were constructed by physically manipulating the environment, by adding and removing relevant items accordingly (Figure 1).^1^ For the seaweed condition, drift seaweed from the upper shore was placed in a natural manner in the appropriate zone. To be ecologically representative of marine litter, in both litter conditions the litter was collected from the sites and consisted of more commonly found items on British shores (MCS, 2012). For public-litter, this included drink cans, plastic bottles, sweet and crisp wrappers, and beach toys such as deflated footballs that could be left on the beach or carried there by winds and rivers. For fishing-litter, typical fishing debris such as rope, fishing nets, broken crates and packaging, and industrial rubber gloves were used. A variety of items were used throughout the images to reduce recognizability of individual pieces of litter, ranging from 4 to 12 items in each shot, covering approximately 7% of the entire image.

A total of 24 experimental stimuli were collated, consisting of six different backdrops from three different sites for each condition. To embed this study in previous research and to decrease the salience of the four conditions, 24 other environmental images were used that represented the six predominantly natural scenes in M. P. White and colleagues’ (2010) study: blue-green, blue-urban, blue-only, green-blue, green-urban, and green-only.

#### Participants and design

The sample consisted of 79 undergraduates from the School of Psychology Participation Pool. Seventy-five percent were women, and the average age was 20 years (*SD* = 3.00).

This study used a one-way within-subject design, whereby each participant rated all 10 conditions: the 4 experimental scenes (clean, seaweed, public-litter, and fishing-litter) plus the 6 other types of natural environments. Following the procedure from Study 1, the order of the images was pre-set. However, to further eliminate order effects, two different orders were created for this study. Participants were randomly allocated to one of these.

#### Measures

Extending Study 1’s measures that looked at the perceived restorative quality of the environment and preference, this study focused further on the restorative potential and outcomes. Images were thus rated according to preference, along with affect and restoration likelihood. Developing the measure from Study 1, two preference items were used on a 10-point scale from *not at all* to *extremely.* Similar to M. P. White and colleagues’ (2010) study, the items *how attractive do you find this view* and *how willing would you be to stay in a hotel with this view* were found to produce a reliable scale (αs > .83). The measurement of affect was based on the Circumplex Model of Affect (Russell, 1980), examining both valence and activation. Participants were asked how the scene made them feel on a scale from *very sad* (1) to *very happy* (10; henceforth *mood*) and from *very calm* (1) to *very excited* (10; henceforth *arousal*; M. P. White et al., 2010). Restoration likelihood for each image was examined with the following question: To what level would you agree with the statement: *I would be able to rest and recover my ability and focus in this environment* on another 10-point scale from *not at all* to *completely* (based on similar studies, for example, Nordh, Hartig, Hagerhall, & Fry, 2009).

The 14-item Connectedness to Nature Scale on a 5-point scale (Mayer & Frantz, 2004) was included to explore the role of participants’ initial bond to the natural environment (α = .77). Standard demographic items were also included (e.g., age and gender).^2^

#### Procedure and analysis

The connectedness to nature measure was completed online the day before the main study.^3^ Upon arrival at the laboratory the following day, participants were seated in front of a computer monitor and were fully briefed. They were then instructed to imagine that *it is a sunny day and you have decided to go for a leisurely walk. After a while you decide to sit down and take in the view. This is what you see . . .* before proceeding onto the picture-rating task. Participants first performed a trial rating on four additional images with each question displayed below the image, before proceeding onto the main task at their own pace. After completing the rating task, participants answered the remaining survey questions before being debriefed and thanked.

For each condition, average responses were calculated for each measure (preference, mood, arousal, and restoration likelihood). The main analyses were not statistically related to demographic factors (e.g., gender and age main effects on preference [*p* = .10 and .32, respectively], mood [*p* = .45 and .19], arousal [*p* = .66 and .38], and restoration likelihood [*p* = .78 and .07]), nor were they found to differ between the two stimuli orders given to participants (for preference, *p* = .79; mood, *p* = .61; arousal, *p* = .96; restoration likelihood, *p* = .31). Consequently, the main analysis consisted of one-way repeated ANOVAs, followed by repeated contrasts to compare the four experimental conditions. To explore the significance of connectedness, 4 (condition: clean, seaweed, fishing-, public-litter) × 2 (connectedness: high, low)^4^ mixed ANOVAs were used, and further one-way repeated ANOVAs examined the experimental conditions in relation to the other six environments.

### Results

#### Differences between clean environments and those with debris

**Figure 2.**
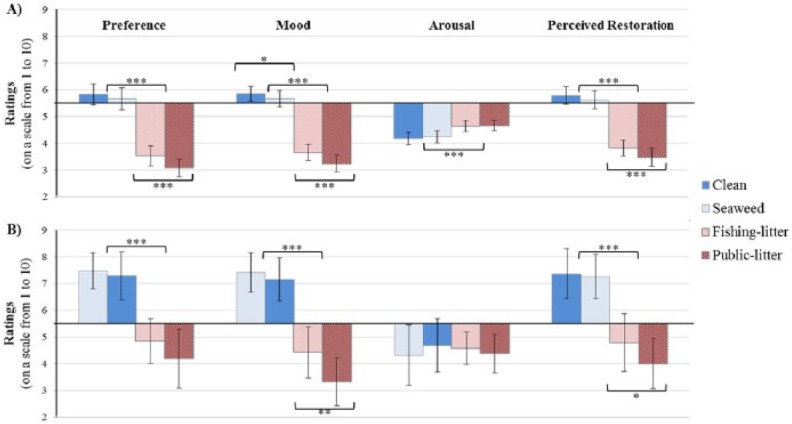
Participants’ average scores (and 95% confidence intervals) for the different coastal conditions centred around the mid-point of 5.5 in Study 2 (*n* = 79; Fig. 2A) and in Study 3 (*n* = 19; Fig. 2B). *Note.* The scale for preference ranged from *not at all* (1) to *extremely* (10); mood from *very sad* (1) to *very happy* (10); arousal from *very calm* (1) to *very excited* (10); and restoration likelihood from *not at all* (1) to *completely* (10). Statistical significance of contrast analyses comparing conditions depicted by * *p* < .05; ** *p* < .01; *** *p* < .001

As shown in Figure 2A, when comparing the four experimental conditions, the same pattern for all four measures emerged: the clean condition was consistently rated most positively, followed by seaweed, whereas the two littered conditions were rated more negatively, with the public-litter condition being rated the worst. In terms of individual measures, participants gave positive preference ratings, felt happy and calm when viewing the clean and seaweed conditions, and perceived them to be restorative environments. In contrast, the two littered conditions were disliked, made participants feel unhappy and less calm, and were seen as less restorative. Statistically, these ratings differed between the four conditions on each measure: preference = *F*(1.39, 108.25) = 190.82, *p* < .001, $\eta_{p}^{2}$ = .71 (large effect); mood = *F*(1.42, 110.89) = 167.21, *p* < .001, $\eta_{p}^{2}$ = .68 (large effect); arousal = *F*(1.74, 135.45) = 13.79, *p* < .001, $\eta_{p}^{2}$ = .15 (small-medium effect); and restoration likelihood = *F*(1.59, 124.19) = 161.79, *p* < .001, $\eta_{p}^{2}$ = .68 (large effect). Repeated contrasts consistently found that ratings were significantly more negative for the fishing-litter compared with the seaweed condition (*p*s < .001), and that public-litter was given significantly different ratings compared with fishing-litter (*p*s < .001; see Figure 2A for all statistically significant repeated contrasts).

#### The influence of connectedness

Overall, the average score of connectedness to nature was 3.24 (*SD* = 0.47 on a 1-5 scale). When including connectedness within the analysis, the main effects of condition remained (*p*s < .001). However, interactions between condition and connectedness were found for preference ratings, *F*(1.41, 108.86) = 5.15, *p* = .02, $\eta_{p}^{2}$ = .06 (small effect), whereby participants with higher connectedness rated the clean and seaweed conditions more positively than did those with lower connectedness. In contrast, everyone was in agreement about littered environments, regardless of the level of connectedness (see Table 2). This interaction also occurred for mood, *F*(1.45, 111.52) = 4.27, *p* = .03, $\eta_{p}^{2}$ = .05 (small effect), and for restoration likelihood, *F*(1.65, 127.05) = 6.45, *p* = .004, $\eta_{p}^{2}$ = .08 (small effect); with the same pattern occurring.

**Table 2.** Participants’ Average Ratings for Clean Versus Debris-Present Coastlines for Respondents With High (*n* = 38) or Low (*n* = 41) Connectedness to Nature in Study 2.

		Preference	Affect–Mood	Affect–Arousal	Restoration likelihood
Condition	Connectedness	*M* (*SD*)	*M* (*SD*)	*M* (*SD*)	*M* (*SD*)
Clean	Low connectedness	5.39 (1.80)	5.59 (1.01)	4.17 (0.79)	5.34 (1.21)
	High connectedness	6.30 (1.53)	6.14 (1.39)	4.18 (1.28)	6.24 (1.60)
Seaweed	Low connectedness	5.26 (1.96)	5.43 (1.28)	4.31 (0.84)	5.15 (1.37)
	High connectedness	6.12 (1.65)	5.91 (1.43)	4.17 (1.23)	6.14 (1.44)
Fishing-litter	Low connectedness	3.50 (1.85)	3.72 (1.45)	4.72 (0.73)	3.76 (1.50)
	High connectedness	3.56 (1.49)	3.57 (1.36)	4.56 (1.02)	3.89 (1.50)
Public-litter	Low connectedness	3.02 (1.70)	3.37 (1.41)	4.76 (0.80)	3.39 (1.36)
	High connectedness	3.14 (1.31)	3.10 (1.32)	4.55 (0.98)	3.55 (1.41)

*Note.* Responses ranged from 1 = *not at all* to 10 = *extremely* for preference; 1 = *very sad* to 10 = *very happy* for mood; 1 = *very calm* to 10 = *very excited* for arousal; and 1 = *not at all* to 10 = *completely* for restoration likelihood.

#### Differences between the experimental conditions and other clean natural environments

To embed these findings into the broader restoration literature, six contrasting natural environments were used as a comparison. For the preference, mood, and restoration likelihood measures, the two littered conditions were still rated the lowest and were the only conditions lower than the midpoint (Table 3). The blue-only and blue-green environments were rated the most positively regarding preference, mood, and restoration likelihood. All environments were found to be rather calming, but participants felt calmer for the green-only and clean rocky shore environments. As before, these ratings were found to differ statistically between the different environments for each measure: preference = *F*(3.94, 306.96) = 2.85.11, *p* < .001, $\eta_{p}^{2}$ = .79 (large effect); mood = *F*(3.94, 307.60) = 280.23, *p* < .001, $\eta_{p}^{2}$ = .78 (large effect); arousal = *F*(3.92, 305.87) = 5.95, *p* < .001, $\eta_{p}^{2}$ = .07 (small effect); and restoration likelihood = *F*(4.48, 349.29) = 184.23, *p* < .001, $\eta_{p}^{2}$ = .70 (large effect; see online appendices for additional contrast analyses).

**Table 3.** Participants’ Ratings of the 10 Environmental Conditions in Study 2 (*n* = 79).

	Preference		Affect–Mood		Affect–Arousal		Restoration likelihood	
	Rank	*M* (*SD*)	Rank	*M* (*SD*)	Rank	*M* (*SD*)	Rank	*M* (*SD*)
Experimental conditions								
Clean	6	5.83 (1.73)	6	5.85 (1.23)	9	4.17 (1.05)	5	5.78 (1.47)
Seaweed	8	5.67 (1.86)	8	5.66 (1.37)	7	4.24 (1.04)	6	5.62 (1.48)
Fishing-litter	9	3.53 (1.67)	9	3.65 (1.40)	4	4.64 (0.88)	9	3.82 (1.50)
Public-litter	10	3.08 (1.52)	10	3.24 (1.37)	2	4.66 (0.89)	10	3.47 (1.38)
Predominantly blue								
Blue-only	2	8.50 (1.07)	2	8.25 (1.07)	5	4.52 (2.03)	1	7.82 (1.33)
Blue-green	1	8.72 (0.88)	1	8.27 (1.03)	8	4.22 (2.05)	2	7.70 (1.44)
Blue-urban	5	6.20 (1.22)	5	6.10 (0.99)	1	4.92 (1.04)	7	5.60 (1.26)
Predominantly green								
Green-only	4	7.60 (1.12)	4	7.43 (1.06)	10	3.95 (1.43)	3	7.41 (1.22)
Green-blue	3	8.22 (1.01)	3	7.71 (1.03)	6	4.26 (1.63)	4	7.18 (1.25)
Green-urban	7	5.70 (1.46)	7	5.70 (1.15)	3	4.66 (0.97)	8	5.52 (1.54)

*Note.* Responses ranged from: 1 = *not at all* to 10 = *extremely* for preference; 1 = *very sad* to 10 = *very happy* for mood; 1 = *very calm* to 10 = *very excited* for arousal; and 1 = *not at all* to 10 = *completely* for restoration likelihood.

## Study 3: Why Does Litter Have an Impact?

With another experimental design, Study 2 was able to compare the psychological impacts of differing types of debris. Participants consistently gave the clean and seaweed condition similarly positive ratings, whereas the two littered conditions were consistently given lower ratings in comparison with both the other experimental conditions and other environments. Notably, the public-litter condition consistently received the lowest ratings. In addition, people high in connectedness to nature gave higher ratings for clean and seaweed conditions than did participants with low connectedness, but, regardless of initial connectedness, everyone rated the littered conditions as similarly detrimental. These findings therefore suggest that it is best when the coast looks natural (either clean or with seaweed), whereas the presence of litter has a detrimental impact on preference, affect, and restoration likelihood. In addition, the extent of this detrimental impact depended on the type of litter. To explore *why* the presence of litter influenced people’s ratings, a third study was conducted. Participants in Study 3 were exposed to only one image from each of the four experimental conditions. Similar to Study 2, participants rated the images but were then asked to explain their ratings, producing rich qualitative data. Moreover, instead of students, we recruited a general public sample. This approach allowed us to examine whether the findings from Study 2 would occur in a broader sample while beginning to unravel the differences between litter types.

### Method

#### Participants and design

The sample consisted of 20 members of the public (similar to Wynveen et al., 2010). Participants were recruited from the University’s paid participation pool, where members received £4 (US$6.70) for participating in the study. One participant was omitted after he disclosed that he had not followed the instructions. Of the 19 remaining participants, just more than half were women (58%), and the average age was 35.79 years (*SD* = 17.13).

Similar to Study 2, a one-way within-subject design was used, but for this study participants only responded to one image from each of the four conditions. The order of presentation for both the condition (clean, seaweed, fishing-, and public-litter) and backdrop (from a selection of four) was fully randomized.

#### Materials and measures

The majority of the materials and measures were kept consistent with Study 2, with only modifications addressed here. To reduce the salience of the main manipulation, four backdrops were selected from Study 2. The rating scales for each picture remained the same, examining preference (αs > .79) and psychological benefits (mood, arousal, and restoration likelihood). To reduce demand on the participants, a shortened version of the Connectedness to Nature Scale (Mayer & Frantz, 2004) was used by selecting the four most highly correlated items from Study 2: *I think of the natural world as a community to which I belong; I often feel part of the web of life; I feel that all inhabitants of Earth, human, and non-human share a common “life force”*; and *like a tree can be part of a forest, I feel embedded within the broader natural world*. Responses ranged from *completely disagree* (1) to *completely agree* (5). The four items formed a reliable scale (α = .80). Additional qualitative items were included, where participants were reminded of their rating for each image and asked “What is it about this scene that made you respond this way and why?” with an open-ended response.^5^

#### Procedure and analysis

The procedure was similar to that of Study 2 but after rating the four images, participants were reshown those images to complete the qualitative aspect, before concluding the study and being debriefed.

The analyses of the quantitative data were identical to those of Study 1, apart from the connectedness analysis. As the sample was too small for between-subject analysis, only correlations were reported to illustrate the general trends. As before, gender (e.g., lack of main effects on preference [*p* = .59], mood [*p* = .61], arousal [*p* = .43], or restoration likelihood [*p* = .78]) and age (*p* = .46, .63, .69, and .34, respectively) had no significant relationships, thus these variables were not analyzed further. For the qualitative data, thematic analysis was used (Braun & Clarke, 2006). As the quantitative findings from Study 2 consistently found that (a) the two natural conditions were different from the littered ones and (b) ratings also differentiated between public- and fishing-litter, the qualitative analysis focused on two specific aspects: (a) why did people respond differently to the natural (clean and seaweed) and littered (fishing and public) environments? and (b) why did people respond differently to the fishing- and public-litter? We used a semantic realist approach that assumes a unidirectional relationship between meaning and language to explore these specific aspects (Braun & Clarke, 2006). The data were initially examined to identify prominent unique themes for the natural and littered conditions, and again for the fishing- and public-litter environments specifically. Themes were then developed and refined over a number of iterations. Analysis was completed by the first author, along with active discussions with the second author. To check interrater reliability, 20% of the qualitative data were randomly selected and coded by two researchers. Cohen’s kappa found satisfactory agreement between coders when focusing on the natural and littered scenes (κ = .76) and when investigating the fishing- and public-litter specifically (κ = 1.00; Landis & Koch, 1977).

### Results

#### Quantitative analysis

Statistically, apart from the arousal measure of affect (*p* = .85), ratings differed significantly between the four conditions: preference, *F*(3, 54) = 25.73, *p* < .001, $\eta_{p}^{2}$ = .59 (large effect); mood, *F*(1.90, 34.15) = 39.71, *p* < .001, $\eta_{p}^{2}$ = .69 (large effect); and restoration likelihood, *F*(1.58, 28.52) = 19.20, *p* < .001, $\eta_{p}^{2}$ = .52 (large effect). For these measures, the two natural conditions (clean and seaweed) were rated positively (consistently above the midpoint), whereas the littered ones were rated negatively, with the public-litter consistently given the lowest ratings. The seaweed condition was given the highest ratings for preference and mood, but the clean condition was seen to be most likely to aid restoration; however, these differences were not statistically significant in the contrast analysis (*p*s > .35; see Figure 2B). However, the fishing-litter was consistently rated differently from the clean or seaweed condition (*p*s < .001), with public-litter often rated significantly worse than fishing-litter (*p*s < .03; Figure 2B).

#### The influence of connectedness

For this sample, the average score of connectedness to nature was 3.70 (*SD* = 0.78; possible range = 1-5). The sample was too small to run the same analysis as in Study 2, consequently exploratory correlational analyses were used. There were no statistically significant relationships between connectedness to nature and the ratings for the four measures on each condition (*p*s > .08; see online appendices for the table of the correlations). However, the sample was very small, and the connectedness theme was also considered in the qualitative analysis that follows here.

#### Qualitative reasons for different ratings

##### Why do people respond differently to the natural and littered environments?

For the question “What is it about this scene that made you respond this way and why?” a number of themes highlighted why the natural and littered conditions were perceived differently. For the two natural conditions (clean and seaweed), comments centered around four themes: evaluative descriptions of the scene, psychological benefits, familiarity, and imagining use (see Table 4 for illustrative examples). *Evaluative descriptions of the scene* addressed the scenes in terms of aesthetics and naturalness (including cleanliness) using either positive or negative valenced descriptions. There were general comments, for example, describing the scene as “nature as it should be,” while others focused on specific elements. Comments were predominantly positive, with the occasional comment expressing a preference for sandy over rocky foreshores. Notably, the lack of rubbish in the scene was emphasized (see Table 4 for examples).

**Table 4.** The Themes With Illustrative Examples of Why People Respond Differently to Natural and Littered Environments and to Fishing- and Public-Litter (Study 3, *n* = 19).

Natural (clean and seaweed)	Littered environment (fishing- and public-litter)	Fishing-litter	Public-litter
**Evaluative descriptions of the scene** *It has a lovely view which is uncluttered and the open sea relates to having an open mind. It is a natural view with no manmade objects in it.* Participant ID: PP114 (Condition: Clean) **Psychological benefits** *The jagged rocks indicate erosion and the natural power of the sea is visible from the waves, which gives me a sense of insignificance when compared to the power of the ocean. This releases tension/stress and improves happiness*. PP120 (Clean)**Familiarity** . . . *this bit of coast looks very familiar to me, I go to places like this to calm and center myself. The rugged natural beauty is inspiring and I love it*. PP113 (Seaweed) **Imagining use** . . . *I could imagine sitting on one of the larger rocks and being really content*. PP111 (Clean)	**Experience-disrupting effects of litter** *The rocky shore towards the sea has a nice view. However, the evidence of rubbish on the sandy beach destroys the image*. Participant ID: PP116 (Condition: Fishing-litter)*Note the flotsam (cans and bottles) and whilst it would be better without it . . . only marginally detracts from the overall beauty*. PP121 (Public-litter) **Environmental consequences of litter** . . . *the plastic doesn’t break down for decades or even hundreds of years*. PP119 (Fishing) **Negative emotions** *Although the day is still pleasant & the sea is blue & calm, the debris left behind makes me feel sad for the environment and what the human race does to it*. PP102 (Public) *It’s sad that a nice beach can be ruined by the waste from humans*. PP110 (Public) **Behavioral response** . . . However, rather than dwell on it, I would simply pick up the rubbish, put it in a bin, enjoy the scene and hope that others might enjoy it too. PP111 (Fishing)	**Lack of intention** . . . *nets from fishing boats that could have been brought in by the sea but not deliberately dropped by people visiting*. Participant ID: PP115 (Condition: Fishing)	**Disrespect for nature** *The rubbish seems much more intrusive this time, people being careless and disrespectful; whereas in the [fishing-litter] scene it could have been brought there more by accident. It gets in the way much more of the enjoyment of the scene* . . . Participant ID: PP109 (Condition: Public) **Physical risks** *Presence of non-biodegradable objects (plastics) is really harmful to ocean life. Furthermore, I would not like to step on the “what looks like muddy area” places and which are full of rubbish*. PP116 (Public) **Reminiscent of the city** *It makes me feel sad as this kind of pollutant should only be seen in the city*. PP105 (Public)

The *psychological benefits* theme consisted of comments that explicitly noted positive effects of the natural scenes on the participant’s state of mind. Many of these focused on experiencing restorative effects and/or an improved mood from viewing these environments, an elaboration of the mood and restoration likelihood ratings. There were also comments that related specifically to feeling connected to nature and how the natural environments were seen to facilitate or inhibit this need. The majority of these comments were positive; however, one participant stated a preference to be close to the sea, therefore the low tidal state was seen to inhibit the full benefit of this environment.

The theme *familiarity* was defined as references to familiarity, feeling at home, and/or reminiscing over happy memories. The sense of familiarity triggered by these natural images was also associated with the psychological benefits. For instance, a couple of responses explained that the main reason to visit this type of familiar environment is to receive those psychological benefits (e.g., see Table 4).

The final theme, *imagining use*, also had links to the psychological benefit theme, where comments spontaneously referred to how people imagine using that environment. Most of these comments expressed more behavioral aspects, picturing engagement in specific activities such as exploring the intertidal area, with other comments also stating anticipated psychological benefits, such as sitting and feeling content.

Overall, the comments referring to the natural environments were mainly very positive and focused on the psychological benefits the environment facilitates, the familiar and positive elements of the scene (such as being clean), and how they could imagine using and experiencing that natural environment. In contrast, the themes for the two littered conditions were much more negative.

Four themes elaborated on why the two littered conditions were rated mostly negatively: experience-disrupting effects of litter, environmental consequences of litter, negative emotions, and behavioral response (see Table 4). Some comments simply noted the presence of litter; however, a prominent theme in the two littered conditions was the *experience-disrupting effects of litter*. This theme emphasized the presence of litter and how it is seen to subtract from the positive aspects of the scene. These comments often included positive descriptions about the surrounding environment but would also emphasize that the presence of the litter “ruins” or “spoils” the scene. The extent of this disrupting effect varied between participants that claimed it “destroys the image” and those that perceived it to “marginally detract from the overall beauty.”

Another theme that explained participants’ responses to the two littered conditions was the *environmental consequences of the litter*. This theme included the anticipated impacts of the litter on the environment spatially (ending up in the sea) and temporally (long-term effects).

Another prominent theme in the two littered conditions was *negative emotions*, with comments explicitly referring to feelings such as sadness and anger as a result of the litter. These negative emotions were often linked to the presence of litter generally but were also associated with the previous themes: feeling sad *because* of the environmental consequences or *because* of the experience-disrupting effects of the litter.

*Behavioral response*, the final theme found in the littered conditions, was relatively positive. This theme referred to the tendency to actively deal with the litter. To eliminate the detrimental effect that litter was seen to have on people’s experiences, some participants noted that they would remove the litter.

Overall, these qualitative data highlighted that the two natural conditions were rated positively because of the psychological benefits they promote, the positive aspects of the scene, that the environment reminded participants of familiar environments, and because they could imagine how they would use and experience that environment. However, the two littered conditions were rated negatively because the presence of litter elicited negative emotions, litter was described as disrupting the benefits the coastal environment typically provides, and respondents mentioned the environmental consequences of those items. However, the impact of litter on the individual seemed to vary between participants, with some overcoming these impacts by picking up the rubbish to enjoy a clean coastline. These litter-related themes were found for both types of litter (fishing and public); however, it was also apparent that there were subtle differences between the two.

##### Why do people respond differently to the fishing- and public-litter?

Even though there was considerable agreement between the two littered conditions, there were additional themes that subtly distinguished the two. A unique theme for the fishing-litter was the *lack of intention*. This kind of response attributed the litter items on the beach to an accident rather than the carelessness of leaving rubbish behind.

Unlike the fishing-litter, the public-litter was seen to be deliberately left. The theme *disrespect for nature* addressed this, with comments emphasizing the individuals responsible for leaving litter, especially their disrespect regarding the natural environment. Many comments described the individuals as acting in a “careless” or “selfish” manner and “disrespecting” nature. This theme was also commonly associated with the earlier *negative emotions* theme, as people expressed anger toward those who were believed to have deliberately littered the environment.

Public-litter was also associated with the theme *physical risks*. This theme addressed the potential consequences of litter for both other people and wildlife. These consequences included dangers to wildlife from mistaking rubbish for food and the risk of potential injury to visitors by standing on items.

The final theme for the public-litter was titled *reminiscent of the city*, which stressed that this type of litter does not belong to the coastal environment and is recognized to be more of an urban issue. This was not a prominent theme; however, it does nicely contrast with the *evaluative descriptions of the scene* theme for the natural conditions, which often noted aspects belonging to nature and how things should be.

In sum, the reason why public-litter was consistently rated more negatively to fishing-litter was found to be centered on the implied deliberateness and disrespect for nature by the litter culprits, the physical risks associated with that specific type of rubbish, and the city orientation of such items, which should only be seen in urban environments.

## General Discussion

While the effects of marine litter on the environment and wildlife are well established, the present research investigated the impact of litter on people. Previous studies have typically focused on green contexts, grouped litter with other degraded features, and directly asked participants about these features. Prior research has rarely examined individuals’ feelings and expected impacts but rather focused on evaluations of the environment. In contrast, this article focused on the global issue of marine litter and carefully took account of actual types of litter evidenced in the marine science literature. By using a subtle yet systematic manipulation where litter was not explicitly stressed to the participants, we adopted a mixed-methodology approach to examine how marine litter influences the perceived restorative quality of an environment and how it may affect people’s experiences. Our three studies present evidence that the presence of litter can undermine the psychological benefits typically provided by clean coastal scenes. Littered coastal environments were seen to have a lower restorative quality, were less liked, and resulted in lower mood and restoration likelihood than did the natural scenes. Study 1 also showed that low tide was perceived as more restorative than high tide, suggesting that less water may be better than more water. Study 2 showed that people high in connectedness rated clean scenes more positively than did people low in connectedness; however, everyone rated the littered scenes negatively regardless of their level of connectedness. In terms of the type of litter, public-related litter had the most negative impacts. Litter was associated with disrupting visitors’ experiences of the natural environment, with detrimental consequences for the environment. The presence of litter elicited negative emotions and yet sometimes provoked a behavioral response to tackle the issue. Public-litter (defined by MCS, 2012, as items that could be left on the beach or carried there by winds and rivers) was seen to be especially bad, as it implied disrespect for nature by other users, had physical risks associated with it, and was seen as belonging to the city.

These findings both support and extend previous work. Mirroring prior studies (e.g., Ashbullby et al., 2013; Hipp & Ogunseitan, 2011; M. P. White, Pahl, et al., 2013; M. P. White et al., 2010), our clean blue environments were rated positively for a range of psychological benefits. We show that restorative quality according to ART (Kaplan & Kaplan, 1989) was rated highly for clean scenes in Study 1 (especially during low tide). The themes from the qualitative data in Study 3 provide further indirect support. For example, the comments that described how participants could use the environment could relate to *compatibility*; the evaluative descriptions of specific elements of the scene that captured the participants’ attention could be associated with *fascination*; the diversity of elements participants focused on and that some could imagine exploring the environment could relate to *extent*; and finally, the joyous reminiscences of past recreational visits and the absence of comments relating to work, stress, and everyday more mundane things could imply that participants received a sense of *being away*. These findings are also reminiscent of the place meaning literature, where studies have found that individuals freely note imagining using the environment (Gunderson & Watson, 2007) and emphasize cleanliness and lack of litter (Davenport & Anderson, 2005; Gunderson & Watson, 2007; Wynveen et al., 2010). It is noteworthy that our research showed very similar themes although we focused on a much less researched environment—temperate coastal environments.

Crucially, our studies provide evidence that litter is a key factor that can undermine the positive effects of clean, pristine coastal environments. While some prior studies have shown similar effects for green environments or when litter was grouped with other features of degradation (Anderson & Brown, 1984; Ballance et al., 2000; Pretty et al., 2005; Tudor & Williams, 2006), we extend these findings to one of the biggest ecological threats—marine litter—and took care to reflect real litter data in our experimental manipulations. It should be noted that these litter manipulations were subtle and only covered a minor area on the photos. Our scenes were thus not comparable with, for example, a coastline after a storm surge or unfavorable wind conditions. Nevertheless, this small amount of litter, which was not explicitly highlighted to the participants, was enough to produce strong and consistent effects. The qualitative data also emphasized the negative emotions associated with litter, which had previously been found within urban park settings (Main, 2013).

This article also begins to explore the reasons for the negative effects of litter. Uniquely relating it to a theory previously applied to more pristine environments, litter was seen to reduce the restorative properties outlined in ART (Kaplan & Kaplan, 1989). This outcome was demonstrated both quantitatively with the lower ratings of perceived restorative quality in Study 1 and qualitatively in Study 3 where the themes could be interpreted to imply that the environment no longer meets the four necessary properties. For example, the emphasis on the presence of litter alone could be seen to distract the viewer from the *extent* and richness that the environment has to offer. These littered conditions could also be seen to lack the same sense of *being away* as the natural conditions, as the litter was seen as a stressor cue, especially for the public-litter, as one participant described it as a city feature. The more detailed responses referring to the physical risks within the public-litter condition, such as standing on the items, implied that the littered scenes were not *compatible* with individuals’ behavioral goals. The *fascination* property could theoretically be seen to have been met as elements of the scene still grabbed the participants’ attention; however, this was not as positive as for the natural conditions, as the focus was on the litter, typically distracting the viewer from the scenic beauty surrounding it. Thus, these new interpretations from Study 3 indirectly support the ratings from Study 1, suggesting some explanation for why littered conditions were not as restorative, yet it is important to stress that this is an insight that requires further testing.

As well as noticeable impacts from litter in general, differences also emerged regarding the more specific debris type. The clean and seaweed conditions rarely differed in their ratings, with similar psychological benefits being reported. In contrast, the two littered conditions did differ, with the public-litter always rated more negatively than the fishing-litter. We showed for the first time that the detrimental effects are not simply due to the presence of marine litter but also the *type* of litter. Some studies have found that when showing people images of specific items, medical waste such as syringes are perceived as especially offensive (e.g., Tudor & Williams, 2003). However, to the authors’ knowledge, the latter two studies presented here are the first to present types of litter based on actual marine litter monitoring data and find that different *sources* of litter have different impacts on people’s experiences. It could be that the public-litter reduced the restorative quality of the environment (e.g., reducing a sense of being away as the items acted as a cue of city stressors); however, this can only be a speculative interpretation as ART’s properties were not explicitly tested when examining litter in this finer detail. However, the qualitative data in Study 3 were able to help identify reasons for the differing impacts. The main explanation that was used to justify the more negative ratings for public-litter was focused on the littering individuals. Participants seemed to focus on the intent of the culprits, with fishing-litter seen as a more accidental by-product of a profession, whereas public-litter was a result of careless visitors not respecting the environment and deliberately leaving rubbish. This finding can be linked to the literature comparing technological catastrophes with natural disasters, where the former are seen as worse and potentially more controversial due to humans being responsible and having a higher level of control over the issue than is true for natural disasters (e.g., Baum, Fleming, & Davidson, 1983).

Another influence on people’s experiences of natural and littered environments is linked to their initial bonds with nature (connectedness to nature) or to a specific location (place attachment). Previous work has shown that people with greater levels of connectedness experience greater psychological improvements from natural (clean) environments than do those with lower connectedness (Mayer & Frantz, 2004; Mayer et al., 2009). We also found that participants with higher connectedness to nature in Study 2 rated the clean and seaweed conditions more positively than did those with lower connectedness. However, this outcome was not replicated in Study 3, possibly due to the smaller and different sample. Some comments within the qualitative data reflected that the natural conditions were rated positively because of the sense of closeness to nature, but the lack of correlations within the quantitative data suggests that connectedness to nature was not a pronounced factor for this sample. In contrast to the natural conditions, the two littered conditions were rated similarly negatively, regardless of connectedness, thus implying that bonds with nature only have an influence on environments when in a pristine state. Of the three perspectives in the place attachment literature; a person’s bond with nature is associated with (a) greater adaptive capacity, (b) greater sensitivity, or (c) not distinctively associated with litter; this uniform response to litter is consistent with the third perspective (e.g., Eder & Arnberger, 2012; D. D. White et al., 2008). Thus, these findings imply that litter is a universal problem in terms of psychological impacts.

### Methodological Limitations and Future Research

These three studies were mainly in agreement; however, some differences were observed. For example, the ratings differed across the studies. These could be due to the different sample sizes and demographics across the studies (e.g., Study 3 was considerably smaller due to the focus on the qualitative component and used a general public sample), the items used (e.g., Study 3 adopted a subset of the original connectedness items that were still statistically reliable, but this may contribute to the differences), and the varying stimuli sets (e.g., Study 2 used a much bigger, more balanced, and diverse sample of stimuli). However, overall the main conclusions remained the same for all studies: natural environments were found to offer beneficial impacts for viewers, whereas the littered conditions undermined these benefits, with public-litter being the worst litter-type.

Some limitations remain. The laboratory approach enabled different variations of coastal environments to be systematically compared. Studies have shown that there is great consistency between findings from laboratory and field studies (e.g., Ryan et al., 2010); however, the findings cannot be generalized to individuals when experiencing these environments in person. For example, more senses are activated when visiting the coast (such as auditory and olfactory senses), which could play an important role in people’s experience when in situ. Future research may also wish to extend this work to larger, more representative samples. Students are popular population for research (e.g., Nordh et al., 2009; M. P. White et al., 2010), but the generalizability of these results can be questioned. We did recruit a general public sample for our last study, which replicated the main conclusions from the previous two, but due to its small size for the qualitative nature of that study, it would be valuable to explore these relationships further in other larger samples. Studies 1 and 2 were novel in that they examined different types of litter. While the majority of the litter commonly found in the U.K. coast was represented, it did not address all categories of litter (MCS, 2012). Therefore, future research may wish to also extend this analysis to other categories and quantities.

Optimistically, future work could focus on tackling this environmental (and, as demonstrated, psychological) issue. Governing bodies have already spent considerable money on removing rubbish from the marine environment (Mouat, Lopez-Lozano, & Bateson, 2010), and legislation has been passed at national and international levels (e.g., the International Convention for the Prevention of Pollution From Ships [MARPOL] Annex V and the EU Marine Strategy Framework Directive). However, even with these efforts, marine litter remains an ongoing problem. Consequently, interventions should focus on the sources of litter. As this article emphasized that the most common source of litter (public-litter) also shows the most detrimental impact on individuals, interventions could be tailored to this specific source. For example, adopting psychological approaches to behavior change, more work could implement both antecedent strategies (interventions prior to the target behavior, such as prompts, social norms, and waste facilities) and consequence strategies (intervention post the target behavior, such as fines and rewards) that discourage littering and encourage removing and disposing of waste responsibly (see Huffman, Grossnickle, Cope, & Huffman, 1995, for an overview). However, it is important to consider the system of actors that contributes to marine litter, including product designers, retailers, consumers, recycling industry, and so on. Only by understanding these different interests and voices will we be able to identify acceptable solutions that reduce the broader problem of marine litter (see, for example, MARLISCO project; www.marlisco.eu).

## Conclusion

While most research on restoration in nature arguably focuses on pristine, clean environments, the reality is increasingly different. Many environments are littered or in other ways damaged or degraded. Thus, it is important to understand the effects such degradation may have on experiencing these environments. Using an experimental laboratory approach that systematically varied photographic stimuli, the present research showed that marine litter can undermine the psychological benefits that the coast ordinarily provides. Coastal scenes with litter were rated negatively in terms of the restorative quality of the environment, and the psychological benefits people derive from them, compared with both clean coastal alternatives and other clean types of natural environments. The intensity of these detrimental effects was found to depend on the type of litter, with public-litter perceived to be the worst. This article begins to apply the ART literature to more degraded environments and adds evidence that marine litter is a substantial problem that needs to be managed appropriately as, in addition to economic and environmental costs, there are also costs to people who visit the coast.

## Supplementary Material

Supplementary material

## Supplementary Material

Supplementary material

## Notes

1. The first author had to justify her littering to other beach visitors a number of times and had to reassure those who approached her that she would remove all items after she had finished.

2. Other items such as environmental experiences, postcode, and upbringing were included but are not central to the current research question, and therefore will not be discussed.

3. This measure was completed prior to the main study to reduce order effects and was done online for the participants’ convenience (there was no control over where the participants undertook this aspect).

4. For ease of interpretation, connectedness was converted to a dichotomous variable by median split into low and high connectedness.

5. Participants’ knowledge regarding the marine environment was also briefly assessed; however, as this is not relevant for this article, it will not be reported.
