# Supplementary material for: Factors That Can Undermine the Psychological Benefits of Coastal Environments: Exploring the Effect of Tidal State, Presence, and Type of Litter
Source: Environ Behav. 2015 Jul 3;48(9):1095–126. doi: 10.1177/0013916515592177 (PMC5066481; doi:10.1177/0013916515592177)
Supplement: Supplementary material [file Supplementarymaterials_1.pdf]

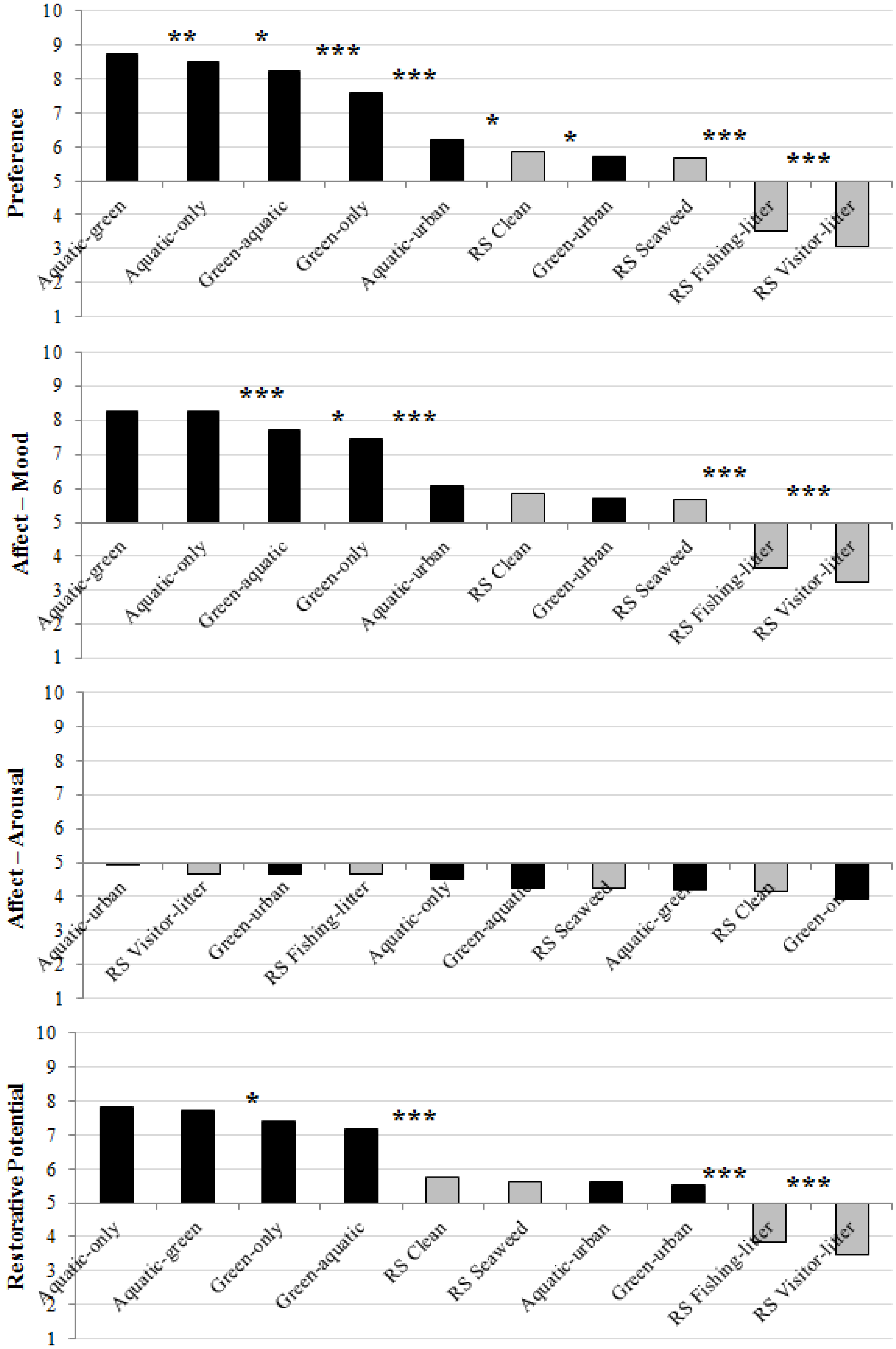


Supplementary Materials 1. *Bar graphs illustrating where statistically significant contrasts analyses were found for the four measures in Study 2 (n = 79; RS = Rocky Shore; * denotes p < .05; ** = p < .01; *** = p < .001).*
