# Supplementary material for: Factors That Can Undermine the Psychological Benefits of Coastal Environments: Exploring the Effect of Tidal State, Presence, and Type of Litter
Source: Environ Behav. 2015 Jul 3;48(9):1095–126. doi: 10.1177/0013916515592177 (PMC5066481; doi:10.1177/0013916515592177)
Supplement: Supplementary material [file Supplementarymaterials_2.pdf]

Supplementary Materials 2. Correlation coefficients between participants' initial connectedness to nature and their ratings of the coastline stimuli for Study 3 (n = 19), separately for each condition.

**Connectedness in Condition**	**Overall Preference**	**Affect – Mood**	**Affect – Arousal**	**Restoration Likelihood**
**Clean**	-.03	.18	.06	.11
**Seaweed**	.01	.07	.28	.02
**Fishing-litter**	.14	.29	.12	.42
**Public-litter**	.01	-.01	.07	.40

Note. None of these correlations were statistically significant (ps > .08).
